# Sports drink consumption and diet of children involved in organized sport

**DOI:** 10.1186/1550-2783-10-38

**Published:** 2013-08-19

**Authors:** Dona L Tomlin, Shannon K Clarke, Meghan Day, Heather A McKay, Patti-Jean Naylor

**Affiliations:** 1 School of Exercise Science, Physical and Health Education, University of Victoria, Victoria, BC, Canada; 2 BC Ministry of Health, 4th Floor, 1520 Blanshard Street, Victoria, BC, Canada; 3 Centre for Hip Health and Mobility, Vancouver Coastal Health Research Institute, VGH Campus. 302 - 2647Willow Street, Vancouver, BC, Canada

**Keywords:** Organized sport, Children, Diet, Physical activity, Sugar-sweetened beverage, Sports drink

## Abstract

**Background:**

Organized sport provides one option for children to be physically active. However, there is a paucity of information about the relationship between children’s participation in organized sport and their diet, and specifically their sports drink consumption. Therefore, the relationship between sports participation in children and the consumption of sports drinks, sugar-sweetened beverages (SSBs) and other components of diet was examined.

**Methods:**

A cross-sectional descriptive study was conducted using baseline data from the Action Schools! BC Dissemination study cohort (n = 1421; 9.90 (0.58) y; 736 girls, 685 boys). The differences between the dietary behaviours of children participating in organized sport (sport) versus those that did not participate (non-sport) was examined. A modified Physical Activity Questionnaire for Older Children (PAQ-C) was used to measure physical activity levels and participation in organized sport. A Food Frequency Questionnaire (FFQ) and 24-hour dietary recall were used to assess eating behaviour and macronutrient intake (including protein, fat, and carbohydrate as well as sugar, fibre and total calories). Fruit, vegetable and beverage quantities were hand-tallied from the dietary recall. Fruit, vegetable and beverage frequency was assessed using the FFQ. Analysis of covariance (ANCOVA) was used to analyse differences between groups and a chi-square test of association was use to determine if participation in sport was significantly associated with the proportion of children consuming sports drinks and SSBs, and with gender.

**Results:**

Children involved in sport had a lower body mass index (BMI) and were more physically active than children in the non-sport group (p < 0.01). Only a small number (n = 20/1421) of children consumed sports drinks and no difference in consumption of sports drink between sport and non-sport participants (p > .05) was observed. However, children involved in organized sport consumed more total calories, fat, fibre, fruit, vegetables and non-flavoured milk (p < 0.01) than non-sport children.

**Conclusions:**

Children involved in organized sport were more physically active, consumed a healthier diet than non-participants and on average had lower BMI’s despite consuming more calories. As consumption of sports drinks among this age group was low, this may be an ideal time to begin educating children and their parents about the appropriate consumption of sports drinks and the perils of consuming too many SSBs, specifically.

## Background

The continued challenge of escalating levels of childhood obesity levels in Canada and around the world demands innovative approaches to healthy eating and physical activity [1]. A healthy diet is a necessary ingredient to promote normal maturation, healthy growth, injury prevention and overall health during the crucial years of growth and development [2]. However, the relationship between nutrition, physical activity, sport, weight management, fitness and health is complex. For example, in theory, children who participate in sport require the highest levels of nutrition to meet the energy demands of their activities. Still, there are limited data that describe the association between sport participation and eating behaviours (including beverage consumption) in children.

Although research that addresses this issue in children is limited, athletic adolescents appear to consume a healthier diet than their non-athletic counterparts [3-5]. Only one study on pre-adolescents [6] was found in the literature and it addressed physical activity rather than sport, demonstrating that increased levels of physical activity in 8–10 year old African-American girls were associated with lower BMI, higher carbohydrate consumption and lower fat intake.

Within the diets of many children and youth, consumption of sugar sweetened beverages (SSBs) has been linked to their excess weight gain [7]. SSBs include carbonated beverages as well as other beverages that contain added caloric sweeteners. Many of these drinks contain few nutrients and excess consumption can also lead to dental erosion and decay [8]. Sports drinks are a specific category of SSBs. Although sports drinks may be helpful in replenishing blood glucose levels during and following high-intensity exercise and maintaining hydration during prolonged exercise in hot environments [9], excessive consumption may increase the risk of children and adolescents becoming overweight or obese [10].

There is limited evidence about the consumption of sports drinks by adolescents and specifically adolescent athletes. Importantly, to the best of our knowledge there are no published data that describe sports drink consumption in children nor specifically about children who participate in organized sport compared to those who do not. In light of the gaps in the literature and with 75% of Canadian children participating in organized sport [11], the purpose of this study was to examine the relationship between sports participation and consumption of sports drinks, SSBs, fruits, vegetables, milk and macronutrients (including protein, fat, and carbohydrate as well as sugar, fibre and total calories) in children.

## Methods

### Study design

A cross-sectional descriptive analysis was conducted using baseline data from the Action Schools! BC Dissemination study, a large cluster randomised controlled trial evaluating the effectiveness of a school-based physical activity and healthy eating intervention (n = 1494). Specifically, the relationship between participation in sport and both eating behaviours (sports drink, SSB, milk, fruit and vegetable consumption) and macronutrient intake (including protein, fat, and carbohydrate as well as sugar, fibre and total calories) in n = 1421 grade 4 and 5 children (9.90 (0.58) y; 736 girls and 685 boys) attending 30 schools across four regions of BC was examined. Baseline data were collected during the fall of 2005. The study was approved by the Clinical Research Ethics Board at the University of British Columbia and the Human Research Ethics Board at the University of Victoria.

### Participants

Students were recruited in September 2005 via invitation/information letters sent home by the teachers. Written consent was obtained from parents/guardians; children gave verbal and written assent. In all, 1494 students consented to participate in the study at baseline. Of those, 1441 students were measured (n = 52 students were absent and n = 1 moved prior to being measured). The 1421 children who completed the question regarding their participation in organized sport that was part of the Physical Activity Questionnaire for Children (PAQ-C) were included in the analysis.

### Measurement procedures

#### Descriptive characteristics

Stretched stature to the nearest 0.1 cm (Seca 214 Portable Stadiometer) and weight to the nearest 0.1 kg (Conair digital electronic scale) were each measured twice and the mean was used in the analysis. Body Mass Index (BMI) was calculated from height and weight as kg/m^2^. Overweight/obesity was calculated using age and BMI [12].

#### Dietary measures

Two instruments were used to assess the diet of each participant. The EHSA Food Processor Nutrition and Fitness Software (v. 10.0, Salem, OR) was used to determine macronutrients (also including total calories, fibre and sugar) consumed from a validated 24-hour dietary recall [13]. As well, fruit, vegetables, milk, 100% fruit juice, sports drinks and SSBs (including flavoured milk, carbonated beverages, non-carbonated flavoured beverages, sweetened coffee and tea, and sports beverages) were hand-tallied from the dietary recall, with serving size determined using the Canadian Nutrient File [14]. Typical frequency of fruit, vegetable, milk and 100% fruit juice consumption was assessed using a targeted Food Frequency Questionnaire (FFQ) adapted from the US National Cancer Institute’s National Institutes of Health: Eating at America’s Table Study Quick Food Scan [15].

#### Physical activity

Physical activity and participation in organized sport was measured using a modified version of the PAQ-C [16]. The PAQ-C is a valid and reliable tool for assessing moderate-to-vigorous physical activity (PA) over the previous 7 days [16,17]. The physical activity score (PA score) ranges from 1 (low active) to 5 (high active) and was calculated from the mean score of nine questions related to frequency and intensity of PA. In addition, students were asked if they participated in organized sport outside of school and then to describe the sport activity and indicate the days they participate in that sport during the week. Those who reported participation in any organized sport and identified the sport and participation frequency were assigned to the ‘sport’ group and those who did not were assigned to the ‘non-sport’ group. Of the students who participated in the study, 886 fell into the sport group (441 girls, 445 boys) and 535 into the non-sport group (295 girls, 240 boys). 65.0% of boys and 59.9% of girls participated in sports (χ^2^ = 3.87, p < 0.05).

### Statistical analyses

Data were analyzed using the Statistical Package for the Social Sciences (SPSS; v20.0, Chicago, IL). Descriptive summaries were generated and the differences in physical activity and dietary measures between sport and non-sport groups were initially analysed using one-way analysis of variance (ANOVA). As there were significant differences in the number of boys and girls between groups and in total caloric consumption, a one-way analysis of covariance (ANCOVA) was used to determine differences in diet between groups while adjusting for caloric intake and gender. A chi-square test of association was used to determine if participation in sport was significantly associated with the proportion of children consuming SSBs or sports drinks, or with gender.

## Results

### Descriptive characteristics

There was no difference in age (p = 0.42) between sport and non-sport groups. However, BMI was significantly lower in the sport group (Difference = 1.65 kg/m^2^, p <0.01) and fewer sport participants were overweight or obese (p <0.01).

### Physical activity

PA score was significantly higher (p < 0.01) in the sport versus non-sport group (Table 1).

**Table 1:** Descriptive characteristics and results of analysis of covariance (ANCOVA) of physical activity, dietary intake and beverage consumption for sport and non-sport children

	**Non-sport group (295 girls, 240 boys)**		**Sport group (441 girls, 445 boys)**		
**Variables**	**N**	**Mean (SD)**	**N**	**Mean (SD)**	**Significance**
***Descriptive characteristics***					
Age (years)	528	9.90 (0.60)	881	9.93 (.57)	p = 0.42
BMI (kg/m^2^)	532	19.96 (3.97)	882	18.31 (3.29)	p < 0.01
% overweight/obese ^a^	532	33.3%	882	27.8%	p < 0.01
***Physical activity***					
PAscore	491	2.9 (0.7)	807	3.3 (0.6)	p < 0.01
***Dietary intake***					
24 hour recall					
Calories (Kcal/d)	527	1837.3 (707.6)	870	1966.8 (755.0)	p < 0.01
Protein (g/d)	527	69.0 (30.9)	870	74.7 (33.0)	P = 0.23
Fat (g/d)	527	62.2 (37.2)	870	65.8 (38.0)	p < 0.05
Carbohydrate (g/d)	527	256.1 (101.1)	870	275.2 (105.8)	p = 0.16
Sugar (g/d)	527	110.8 (58.6)	870	122.0 (64.2)	p = 0.11
Fibre (g/d)	527	14.8 (7.6)	870	16.4 (8.8)	p < 0.05
Fruit servings/d	527	2.4 (2.5)	868	2.8 (2.8)	p < 0.05
Vegetable servings/d	527	1.8 (1.9)	868	2.1 (2.1)	p < 0.05
FV servings/d	527	4.2 (3.4)	868	4.9 (3.8)	p < 0.01
FFQ					
Fruit (times/d)	533	1.1 (0.7)	878	1.3 (0.7)	p < 0.01
Vegetable (times/d)	532	1.0 (0.6)	876	1.2 (0.7)	p < 0.01
***Beverages***					
24 hour recall					
Non-flavoured milk (mls/d)	527	296.2 (298.7)	868	350.8 (332.8)	p < 0.05
100% juice (mls/d)	527	170.1 (249.5)	868	201.0 (269.6)	p = 0.11
100% juice servings/d	527	1.4 (2.0)	868	1.6 (2.2)	p = 0.11
Sports drinks (mls/d)	5	412.0 (236.9)	15	338.3 (230.8)	p = 0.92
SSB - no 100% juice (mls/d)	527	216.9 (285.1)	868	206.9 (306.3)	p = 0.11
SSB - with 100% juice (mls/d)	527	387.0 (357.4)	868	407.9 (385.4)	p = 0.88
FFQ					
Milk (times/d)	535	1.3 (1.2)	885	1.5 (1.5)	p < 0.05
100% Juice (times/d)	535	0.8 (1.0)	882	0.9 (1.1)	p < 0.05

^a^ – determined by Cole [12].

*FV* = Fruit and vegetable.

*SSB* = Sugar sweetened beverage.

### Dietary measures

Results from the 24-hour dietary recall and FFQ are provided (Table 1). Total calories and gender differed significantly between groups. When controlling for these the sport group consumed significantly more fibre, vegetable and fruit servings (independently and together) and non-flavoured milk, but a similar amount of protein, carbohydrate and sugar compared with the non-sport group. From the FFQ, the sport group consumed fruit, vegetables, non-flavoured milk and 100% juice more frequently than the non-sport group. Consumption of SSBs or sports drinks did not differ significantly between the groups. Similar proportions of sport and non-sport participants reported SSB (χ^2^ = .626, p = .429) and sports drink (χ^2^ = 1.38, p = .240) consumption on the dietary recall.

## Discussion

The profile of children participating in organized sport compared to those that were not provides new insight into the relationship between sport participation and children’s consumption of sports drinks specifically, and aspects of their overall diet generally. Contrary to previous reports on adolescents no difference was found in consumption of sports drinks or SSBs between children participating in sport and those that were not. However, similar to previous reports, children involved in sport had, on average, lower BMIs, were more physically active and had a healthier diet profile (consumed more fruit, vegetables, non-flavoured milk and fibre). Each of these will be discussed in turn.

### Descriptive characteristics

BMI is considered by some to be a reasonable measure of adiposity in children [18]. This study adds to a small body of literature that investigated the relationship between sport participation and BMI in children. Based on BMI, higher proportions of overweight and obesity were seen in this study (29.8% overweight or obese) compared to Canadian children measured in the 2004 Canadian Community Health Survey (CCHS; 25.8% overweight or obese) [19] but in the present study the sport group had lower BMI (18.31 versus 19.96 kg/m^2^; p < 0.01) and lower rates of overweight/obesity (27.8 versus 33.3%; p <0.01) than the non-sport group.

These findings align with a few studies that reported that organized sport participation in children was associated with lower BMI [6,20,21] while contradicting other findings that found no association between sport participation and weight status [22]. The different methods adopted across studies might partially explain these variable findings. One study used an overweight cut-off point [21] as was used in the present study, and another used an obesity cut-off point [22]. For analysis some studies calculated simple correlations [6,20] while the present study applied ANCOVA to evaluate group-based differences.

### Physical activity

While 62.4% of children in the current study reported participation in organized sport, the Canadian Fitness and Lifestyle Research Institute (CFLRI) reported 75% sport participation among 5–17 year old Canadians [11]. This discrepancy may be because while the CFLRI results were based on parental reports, children involved in the current study self-reported their participation. The results are also consistent with previous findings that children involved in organized sport are more likely to be physically active than non-participating peers [22,23]. The PA score averages of 2.9 and 3.3 for non-sport and sport groups, respectively are similar to those reported in grade 4, 5 and 6 students in the United Kingdom [24] and 9–18 year olds in Canada [25].

### Dietary measures

The healthier diet profile observed in the sport group was consistent with previous research on adolescent athletes who, on average, consumed significantly more health promoting foods such as milk and fruit [3,4,26] and, for boys, more vegetables as well [26]. The sport group had higher caloric intake, consuming more fruit, vegetables, fibre and non-flavoured milk than the non-sport group. Even so, less than 50% of the children in sport and non-sport groups met recommended guidelines for fruits and vegetables and the sport group consumed more fat. While these results support the notion that sport-involved children have healthier diets, clearly the diets of both groups have room for improvement.

SSB consumption by both sport and non-sport children in the study was slightly lower than the 450–534 g reported for 9–13 y olds in the CCHS [27]. As well, unlike other reports on adolescents, no differences in SSB or sports drink consumption was observed between those who were and were not involved in organized sport. Ranjit and colleagues noted a positive association between sports drink consumption and participation in organized physical activity and a negative association between soda consumption and organized activity in adolescents [10]. In other research, athletic adolescents were more likely to consume sports drinks than non-athletic adolescents [3]. It is possible that the younger cohort in the current study was not yet influenced by coaches and the media, or was not involved in high intensity training and sport competition (back-to-back training, multiple games or tournament play). It may also be that the younger students lacked the purchasing power of the older adolescents.

### Strengths and limitations

One novel element of the study was that, to our knowledge, it is the first examination of sports drink consumption in this age group. A strength of the study was the relatively large sample size (n = 1421) of similar aged children. Also, two different instruments were used to assess diet and even though the dietary recall measured volume and the FFQ measured frequency, both instruments showed similar trends.

We also acknowledge that a cross-sectional study has a number of limitations. Self-report instruments were used to assess PA and diet. In an effort to mitigate this limitation, a questionnaire (PAQ-C) with well-established internal reliability and validity [16] was employed. The measure of organized sport did not address the quality of participation (e.g. intensity) or seasonal variations in level of participation. A single 24-hour dietary recall was used which, despite its common usage, has been criticized for not capturing usual patterns of food consumption [28]. This is especially true for food and beverages, like sports drinks, that may not be consumed daily. Finally, the cross-sectional nature of the data prohibits an evaluation of any causal relationships between variables.

## Conclusions

This data suggest that pre-adolescent children involved in sport have healthier diets, physical activity and weight profiles (despite consuming more calories) than children not involved in organized sport. It also shows that at around age 10 years only a small proportion of children are consuming high calorie sports drinks. This speaks to the pre-adolescent period as a potential ‘window-of-opportunity’ when parents and coaches might exert a positive influence on children’s behaviour regarding consumption of sports drinks and SSBs. Athletes and their parents should be educated about proper nutrition relative to their child’s/athlete’s level of training and competition; including the dangers of high ‘empty’ nutrient value of SSBs and sports drinks.

## Abbreviations

ANOVA: One-way analysis of variance; ANCOVA: One-way analysis of covariance; BMI: Body mass index; FV: Fruit and vegetable; PA: Physical activity; PA Score: Physical activity score; SSB: Sugar-sweetened beverage.

## Competing interests

The authors declare that they have no competing interests.

## Authors’ contributions

SC developed the research question, conducted the preliminary analysis and edited the manuscript. DT supported data collection, provided quality assurance and database management, conducted the secondary analyses, then wrote and edited the overall manuscript. PJN and HMK wrote the funding proposal, managed the implementation of the overall study and edited the manuscript. PJN helped develop the research question and also supervised the analysis of the data. MD worked with PJN and HMK to design the healthy eating component of the trial, including instrument selection and analysis and edited the manuscript. All authors read and approved the final manuscript.
